# The Limitations of Abdominal Surgery

**Published:** 1905-04-15

**Authors:** D'Arcy Power

**Affiliations:** Surgeon to St. Bartholomew's Hospital and to the Bolingbroke Hospital, Senior Surgeon to the Victoria Hospital for Children, Chelsea


### THE LIMITATIONS OF ABDOMINAL SURGERY.

By D'Arcy Power, F.R.C.S.Eng., Surgeon to St. Bartholomew's Hospital and to the Bolingbroke Hospital, Senior Surgeon to the Victoria Hospital for Children, Chelsea.

The whole duty of an abdominal surgeon is summed up in a knowledge when to operate and when to refrain from operating. He may fall into two great errors. He may await developments when it is imperative that he should operate at once, or he may operate when time and ordinary remedies would have been sufficient to cure his patient. The abdomen is full of traps and pitfalls, not only to the unwary and to the beginner, but even to those who have the greatest experience. The mere matter of diagnosis, therefore, is of secondary importance if the knowledge of when to operate and when to abstain has been thoroughly ground into a man. In abdominal surgery, as in other conditions, it is the borderland and anomalous cases which are difficult to recognise and treat, where, for some reason, the symptoms are masked or the disease does not run its ordinary course. In other cases a mistake is made either on account of the rarity of the condition or because preconceived ideas are entertained which in actual practice are found to be inaccurate. Instances of both these causes of error are easily adduced. Within the last year a girl was admitted into the Victoria Hospital for Children with a diagnosis from the general practitioner of appendicitis, the diagnosis being based on the fact that there had been a little pain in the right iliac fossa with some tension of the abdominal muscles. I examined the patient immediately after admission and found slight rigidity, but there was no rise of temperature and no accleration of pulse. On the next day the pulse and temperature fell to normal. For a week I saw the patient at intervals and she was constantly under the observation of a competent house-surgeon, whose only complaint was that he could not get her bowels open. On the eighth day she died suddenly, and at the post-mortem examination the appendix was found to be gangrenous. I blamed myself for not operating, and I remembered the case. A few weeks ago I was summoned to see a girl who had been ill for a few days with abdominal symptoms, but without any great rise of temperature, and with very slight quickening of the pulse; bearing the last case in mind, I opened the abdomen at once and found a perforated appendix lying in an abscess. The pus in the abscess was nearly odourless, and the patient made a speedy recovery. In both these cases it would seem as though some want of bacterial toxins had produced the modified symptoms.

The rarity of the disease often leads to errors in diagnosis and to consequent delay in treatment. A busy practitioner, when he is in the middle of an outbreak of diarrhœa, may easily overlook a case of intussusception. He will recognise a perforated gastric ulcer in a woman, but he may at any time fail to recognise a perforated duodenal ulcer in a man, because he has not hitherto seen one, and he has still to learn that the symptoms are referred by the patient to the right illic fossa rather than to the seat of perforation. It is probable, indeed, that in neither case does the question of intussusception or of perforation ever occur to him unless the signs are unusually well marked. He simply treats the patients on the ordinary lines, and it is only when his remedies have failed or the patient gets visibly worse that he is driven to look for other causes.

The surgeon, on the other hand, is equally handicapped. He does not see the countless cases of colic, of premenstrual pain, or the vague but alarming abdominal symptoms which sometimes usher in pneumonia and influenza, and if by chance he comes across such a case he attributes a much greater importance to the symptoms than they in any way deserve. Even in the graver cases the diagnosis is often made easier for him than for his colleague in general practice, because time has elapsed and he has the advantage of hearing the opinions of several witnesses before he arrives at his own conclusion.

Speaking for myself I have very little doubt that I am guided to operate or not to operate very largely by the general condition and aspect of the patient, bearing in mind the Hippocratic axiom in the book on Prognostics. "He should observe in acute diseases first the countenance of the patient." Even if he be in considerable pain in unimportant conditions he never has the drawn and anxious expression of a patient who has severe abdominal mischief. A first glance, therefore, is often enough to reassure one and to make one feel that there is nothing very serious the matter. But many pitfalls attend this method of prognosis by simple observation. A child with a large and acute intussusception may lie placid and lethargic, whilst another child with a much smaller invagination may present the classical symptom of acute pain. A man with a perforated duodenal ulcer passes through a period of comparative relief between the subsidence of the acute symptoms of perforation and the appearance of the inevitable peritonitis. A patient with an attack of appendicitis may actually seem better after the appendix has become gangrenous or when it has ruptured than whilst there was simple inflammation. Too much reliance, therefore, must not be placed upon the appearance. It is useful as a clue, but it must be made to fit in as a part of the general puzzle, and not used to the exclusion of other means of diagnosis.

The pulse is the next and most important guide in conditions of acute abdominal mischief. In children and in very nervous people it is useless, but in a large number of cases it gives the most valuable information as to the progress of intra-abdominal inflammation. Thus if the pulse remains quick, or increases in rate, the prognosis is bad, even though the general condition of the patient is thought to have undergone material improvement. In acute abdominal conditions therefore I always ask that the pulse shall be counted by the same person every half hour until I am able to see the patient; information is thus obtained of great value and free from the disturbing influence of nervousness.

The temperature is of much less value than either the appearance or the pulse, and it is of even less value in children than in adults. The thermometer is actually misleading in many cases of abdominal perforation if its readings be adopted without correlating them with the other indications. In cases of appendicitis it is too often said by the friends, and even by the doctor, that the patient is decidedly better because the temperature has fallen and the patient has passed a good night, being freer from pain, when in reality perforation or gangrene of the bowel has occurred, as might have been known if the pulse had been counted and the abdomen had been examined.

The physical signs far outweigh the appearance, the pulse, and the temperature in a case of acute abdominal disease, though even these are sometimes fallacious. Inspection, palpation, and percussion all yield information of great importance in such a case. We learn by inspection whether the abdomen is moving freely during respiration; if it be not moving freely, whether the limitation of movement is general or confined to any one part, whether there is a general distension or whether the muscles on one or both sides are in a state of tonic contraction. Palpation conducted with the utmost gentleness and begun over the left iliac fossa, the hand being carried over the abdomen in the course of the large intestine may show that the whole abdomen is rigid and contracted or rigid and distended. When it is rigid and contracted isolated tender spots will usually be manifest, perhaps, over the region of the stomach or to the left of the umbilicus, but when the whole abdomen is rigid and distended the pain is usually generalised in acute conditions because there is general peritonitis. The rigidity may be limited to the right iliac fossa which the patient indicates as the seat of maximum pain both in appendicitis and in perforated duodenal ulcer as well as in torsion of the right ovary or ovarian cyst. Percussion may show that the liver dulness has wholly disappeared or is markedly reduced when the abdominal walls are rigidly contracted. I do not think it is of much importance, for the liver dulness disappears when there is no free gas in the peritoneal cavity in cases where, from some cause or another, there has been acute dilatation of the stomach, and I have seen it disappear and reappear again in the course of a few hours. General tympanites is of much worse import. It means paralysis of the intestines due to septic absorption associated with peritonitis, and is only too often the sign of a fatal ending to the case. Too little use is made of rectal examinations as a routine method of procedure in the more difficult cases of acute abdominal mischief. Information of the greatest value can often be obtained in this manner in cases of acute intussusception, of appendicitis, and in some of the rarer forms of hernia.

Turning now from the general symptoms let us consider the individual conditions to ascertain the mistakes which are likely to be made and the best means of escaping them.

We may begin, according to age, with intussusception, as it is one of the most common causes of acute abdominal trouble in children coming under the notice of a surgeon. The sudden onset of acute abdominal pain with the passage of blood-stained mucus by a child who has been previously in good health affords grave reason for suspecting that it has an intussusception, and this suspicion is converted almost into a certainty by the presence of an abdominal swelling which is often better felt per rectum than through the abdominal walls.

There is but one treatment for an intussusception, and that is the immediate opening of the abdomen and the reduction of the invaginated bowel. It is not worth while to waste time in vain efforts at reduction by inflation or irrigation. Children who are operated upon early usually recover, those who are allowed to wait generally die.

Mistakes arise in at least three ways in cases of intussusception. Suspicion is excited by the symptoms that the child is suffering from an intussusception, but an operation is delayed because no tumour is felt in the abdomen. It is not difficult usually to feel the invaginated bowel, because the abdomen remains supple; but the invagination may be quite small, and yet acute and deadly as in the enteric forms, or it may not be apparent to a casual examination. A little patience will generally meet with its reward, for a swelling which is imperceptible at one time is quite easy to feel a few minutes afterwards. Another cause of mistake, I have already mentioned, that the child may be so lethargic as not to be suffering acutely, and it is hard to believe that he has an intussusception at all. These cases are generally found to occur in large intussusceptions, where, either from loss of tone in the muscles or from the disproportionately large size of the colon, the small intestine has slipped easily into the large intestine.

I recall at least two cases in which I have opened the abdomen to relieve an intussusception, and have found other causes for the symptoms. In one there was an intussusception of the large intestine into itself, but the tumour, which had been felt before the operation, was found to be due to a mass of enlarged glands at the illeo-cæcal junction. The intestine at this spot had been pulled upwards towards the liver by the inflammation of a previous intussusception which had been successfully reduced by operation a few months previously. In another case the symptoms of acute obstruction in a young child were produced by the adhesions of an old attack of appendicitis, which had constricted the ascending colon just beyond the illeo-cæcal valve.

Perforated gastric ulcer is now a well-recognised condition, and immediate suture is the appropriate treatment for the acute form. The doubtful and difficult cases, from the point of view of the surgeon, are those where the ulcer is leaking and the patient only shows the slighter symptoms of pain and localised tenderness; the difficult cases from the point of view of the general practitioner seem to be those of acute perforation in men. He does not yet realise sufficiently that a gastric ulcer may perforate in men nearly as often as in women. He is, therefore, a little inclined to wait too long when a man presents himself with symptoms of acute perforation for which he would unhesitatingly call in a surgeon at once if the patient had been a woman. In the case of leaking ulcers I prefer to wait when the patient can be carefully and continuously watched. Such leakage usually takes place from an ulcer on the posterior wall of the stomach and is often cured without surgical interference, whereas if the surgeon interferes too hastily he may easily make the patient worse instead of better.

Perforated duodenal ulcer is a much more serious condition than an acutely perforated gastric ulcer. A very large proportion of cases occur in men and personally I have not yet seen one in a woman. The condition, too, is more difficult to diagnose because the patient rallies from the initial shock and either does not seek advice or is content with local applications until the onset of peritonitis compels a closer investigation of his abdomen. When the patient is seen immediately after the perforation the local conditions make the diagnosis fairly clear to those who have seen similar cases. There is marked local tenderness with rigidity on the right side of the abdomen above the umbilicus. The tenderness remains, but the rigidity passes off in a few hours and the patient after a varying time complains of pain in the right iliac fossa which feels full when it is examined. The condition, therefore, is nearly always diagnosed as being due to appendicitis and in many cases a still further delay is recommended. This delay is usually fatal, for it is a peculiarity of duodenal ulcers which have perforated that they allow of the continuous escape of a clear alkaline fluid, which is probably the secretion of Brunner's glands. This fluid escapes constantly into the peritoneal cavity, and if time be given it a very general peritonitis is set up which sooner or later is purulent because the secretion is contaminated with septic products at the point of ulceration. It is of the utmost importance therefore to operate at the earliest possible moment in cases of suspected duodenal ulceration, but even when the perforation is found it lies so deeply under cover of the left lobe of the liver that it is no easy matter to close it effectually.

There are other conditions which simulate acute intestinal perforation and yet are much less amenable to surgical treatment. Foremost amongst these are acute inflammation of the pancreas and sudden thrombosis of the portal vein. Acute inflammation of the pancreas is to be suspected when a person who has been previously in good health, or who has suffered from comparatively trivial symptoms of indigestion, is seized suddenly with a violent pain in the epigastrium, followed by vomiting and collapse. The symptoms become less alarming after recovery from the first shock, and in the course of 24 hours a circumscribed swelling appears in the epigastrium which is tympanitic and resistant. This may be associated with general tenderness over the region of the pancreas and there are often tender spots at different points in the abdomen. The discovery of patches of fat necrosis when an abdominal section has been performed confirms the diagnosis though their absence does not prove the pancreas to be healthy.

Chronic pancreatitis sometimes leads to thrombosis of the portal vein and its tributaries, and in this way may produce symptoms of acute intestinal obstruction due to pylephlebitis. But the pain in these attacks is much more widely diffused than in acute perforation, and abdominal exploration may reveal inflamed or even gangrenous intestine.

It is clear, therefore, that the various forms of acute intestinal obstruction afford a constant field for the exercise of the diagnostic powers, and whilst some cases are clear enough from the beginning, the causes of others are only revealed by abdominal section,or even in the post-mortem room. Every one is now aware of the singular manner in which acute intestinal obstruction is the predominant symptom in some forms of suppurative peritonitis. Such a case came under my notice not very long ago A schoolboy, aged six, who had always been delicate, vomited and suffered from increasing intestinal obstruction for a week. The abdomen was painful and full, but it was not markedly distended. The pain was limited to the left half of the abdomen, though the left rectus abdominis was less rigid than the right muscle. I opened the abdomen and found a suppurative peritonitis limited to the left side. The boy ultimately made a good recovery. A gall-stone impacted in the small intestine is another cause of acute intestinal obstruction which it is especially hard to recognise. The impaction often occurs in stout and elderly women who have had such obscure symptoms of biliary colic, that only when the gall-stone is removed from the intestine is it possible to correlate the previous symptoms with the true cause. Of such a case I had the following example. A stout lady, aged 68, had complained for two or three years of indefinite abdominal pains which were very severe at times. They recurred at irregular intervals, and during their occurrence the abdominal walls were extremely tender to the slightest touch, although repeated examinations failed to show any point of maximum tenderness or any lump. There was no history of biliary colic nor had the patient ever been jaundiced. She complained of dyspepsia, and she had lost weight. One morning she was suddenly seized with an attack of vomiting and severe abdominal pain referred to the left lumbar and iliac regions. The abdomen was flaccid and moved freely during respiration, and the vomiting soon became fæcal. Within 12 hours of the attack I opened the abdomen in the median line above the umbilicus, as the obstruction seemed to be high up in the small intestine. On raising the great omentum a piece of collapsed small intestine presented itself, and close by it a piece of distended small intestine. The actual seat of obstruction was soon found by tracing the intestine from these two points, and it was found to be due to a spheroidal gall-stone measuring seven-eighths of an inch in its longest diameter. The bowel was closed after the gall-stone had been removed, but the patient died 12 hours afterwards.

The limitations of abdominal surgery at the present time are due rather to errors of diagnosis than to the difficulties of technique. When the condition is recognised early and subjected to operative interference the results are much more satisfactory than when lapse of time has allowed the pathological condition to reach its acme. The difficulties of diagnosis increase necessarily with the rarity of the lesion, but the rarity of the lesion decreases with the experience of the observer. A tumour of the spleen, which would be difficult of diagnosis to one who practised in England, would be easy to a man well versed in tropical medicine; whilst a perforated duodenal ulcer readily to be recognised by a surgeon in London might be very difficult for a man whose whole experience had been in India. That immediate operation does not always save life in acute abdominal conditions is due to the fact that many persons live almost at the end of their powers of resistance—a condition well expressed by the classical authors as the *ætas capularis*, when the hold on life is so feeble that a very small thing determines a fatal issue. It is impossible, therefore, to predict the result of an attack of acute abdominal inflammation. A delicate woman who has seemingly very slight rallying power will come through such an ordeal triumphantly whilst a man who is, to all appearance, healthy, and has never known a day's illness, will die out of hand. Much, however, can be done to prolong the life even of these patients by tiding them over the period of intense shock which proves so fatal. In common with other surgeons, I have used of late years massive injections of saline solution—that is to say, I have introduced an aspirating needle into the subcutaneous tissue of the axilla or Scarpa's triangle—and allowed normal saline, at a temperature of 100° F. to flow in at the rate of a pint an hour until 18 or 20 pints have been infused. The immediate result in every case has been most satisfactory, but in two cases the patients have died subsequently with œdema of the lungs. I am convinced, therefore, that the method is not entirely free from danger, but in desperate cases it is well worth trying, and when the collapse and lowered blood-pressure are very marked the immediate results are of the utmost value.

